# Pembrolizumab Plus Chemotherapy Versus Chemotherapy Monotherapy as a First-Line Treatment in Elderly Patients (≥75 Years Old) With Non-Small-Cell Lung Cancer

**DOI:** 10.3389/fimmu.2022.807575

**Published:** 2022-02-14

**Authors:** Zhengyu Yang, Ya Chen, Yanan Wang, Minjuan Hu, Fangfei Qian, Yanwei Zhang, Bo Zhang, Wei Zhang, Baohui Han

**Affiliations:** Department of Pulmonary, Shanghai Chest Hospital, Shanghai Jiao Tong University, Shanghai, China

**Keywords:** pembrolizumab, chemotherapy – oncology, non-small-cell lung cancer, first line therapy, elderly patients

## Abstract

**Objective:**

Several trials have shown that pembrolizumab plus chemotherapy was more effective in patients with advanced non-small-cell lung cancer (NSCLC) than chemotherapy monotherapy. However, whether pembrolizumab plus chemotherapy is still a better choice for first-line treatment in elderly patients (≥75 years old) remain unknown. We retrospectively compared the efficacy and safety of these two treatments in elderly patients.

**Patients and Methods:**

We collected data of 136 elderly patients with advanced NSCLC who were treated with pembrolizumab plus chemotherapy or chemotherapy monotherapy in our hospital from 2018 to 2020. We compared the progression-free survival (PFS) and overall survival (OS) of patients and analyzed which subgroups might benefit more significantly from pembrolizumab plus chemotherapy.

**Results:**

In total population, pembrolizumab plus chemotherapy showed superior PFS and OS than chemotherapy monotherapy (PFS: 12.50 months vs. 5.30 months, P<0.001; OS: unreached vs. 21.27 months, P=0.037). Subgroup analysis showed patients with positive PD-L1 expression, stage IV, good performance score (ECOG-PS <2), fewer comorbidities (simplified comorbidity score <9) or female patients had demonstrated a more evident OS benefit in pembrolizumab plus chemotherapy. In terms of safety, the pembrolizumab plus chemotherapy group had higher treatment discontinuation (26% vs. 5%).

**Conclusions:**

Elderly patients using pembrolizumab plus chemotherapy achieved longer PFS and OS, but were more likely to discontinue due to adverse effects, so disease stage, PD-L1 expression, ECOG-PS and comorbidities should be considered when selecting first-line treatment.

## Introduction

In recent years, immunotherapy, represented by PD-L1/PD-1 inhibitors, has developed rapidly, leading to a greatly improved prognosis for patients with non-small-cell lung cancer (NSCLC) without targetable oncogene alterations (1–4). Previous studies have shown that chemotherapy had immunomodulating effects enhancing the immunogenicity of tumors, thereby increasing the clinical benefit from immunotherapy (5, 6). Based on the Keynote series, Pembrolizumab plus chemotherapy (P+C) has been approved in China as first-line therapy for advanced NSCLC, regardless of PD-L1 expression (7–9).

It is widely believed that age-related decline of the immune system or immune senescence may lead to poor efficacy of immunotherapy in elderly patients (10, 11). A real-world study in Japan showed that using the same P+C treatment, elderly (≥75 years) patients with NSCLC had significantly shorter progression-free survival (PFS) and overall survival (OS) than younger population (age <75 years) (PFS: HR=2.30 P=0.004; OS: HR=4.58 P<0.001) (12). Thus, our study aimed to explore whether P+C was still the best choice for first-line treatment in elderly patients.

In this study, we evaluated the efficacy and safety of pembrolizumab plus chemotherapy as a first-line treatment in elderly patients and analyzed which subgroups might benefit more significantly.

## Materials and Methods

### Study Subjects

This is an observational and retrospective study. Elderly patients who met all the following criteria from January 2018 to December 2020 were included. First, they were diagnosed with advanced NSCLC (TNM stage IIIB, IIIC or IV) without targetable oncogene alterations (EGFR/ALK/ROS-1). Second, they received chemotherapy or pembrolizumab plus chemotherapy as first-line treatment and had complete follow-up data in our hospital.

Baseline characteristics of the enrolled patients, including age, gender, tumor-nodal-metastasis (TNM) stage, performance score (ECOG PS), histology, tumor proportion score (TPS), comorbidity, and treatments were recorded. The simplified comorbidity score (SCS) system was used to classify patients (13), including smoking history, diabetes mellitus, renal insufficiency, respiratory comorbidity, cardiovascular comorbidity, neoplastic comorbidity and alcoholism.

### Detection of Programmed Death Ligand 1 Expression

Tumor samples were obtained by tissue biopsy at the time of disease diagnosis. PD-L1 protein expression was detected by the PD-L1 IHC 22C3 pharmDx assay and calculated by tumor proportion score (TPS), the percentage of viable tumor cells showing partial or complete membrane staining. It was classified into negative (TPS<1%) and positive (TPS≥1%).

### Treatment and Follow-Up

The monotherapy group was administered chemotherapy including pemetrexed, gemcitabine, taxanes and vinorelbine, with or without platinum. The combination therapy group was given pembrolizumab combined with chemotherapy until disease progression or unacceptable toxicity occurred. Adverse events (AEs) were graded by each physician according to the National Cancer Institute Common Terminology Criteria for Adverse Events version 4.0.

Bases on the Response Evaluation Criteria in Solid Tumors (RECIST v1.1), patients were clinically evaluated every 6 to 8 weeks. The objective response rate (ORR) was defined as the proportion of patients with a confirmed complete or partial response, while the disease control rate (DFS) was defined as the proportion of patients with a confirmed complete or partial response or stable disease. Progression-free survival (PFS) is defined as the time from the initiating first-line treatment to the occurrence of disease progression or the last follow-up. Overall survival (OS) is defined as the time from the initiating first-line treatment to death or the last follow-up. The last follow-up time was July 23, 2021.

### Statistical Analysis

Chi-square test and Fisher’s exact test were used to compare categorical variables and continuous variables between groups as appropriate. The Kaplan-Meier method and log-rank test were used for PFS and OS analysis to compare the prognosis of different groups. Cox proportional hazard models was used for univariable and multivariate analysis to evaluate the variants affecting PFS and OS. A *P* value of less than 0.05 was considered statistically significant. All analyses were performed on the Statistical Package for Social Science (SPSS, Chicago, IL version 22.0).

## Results

### Clinical Features

**Figure 1:**
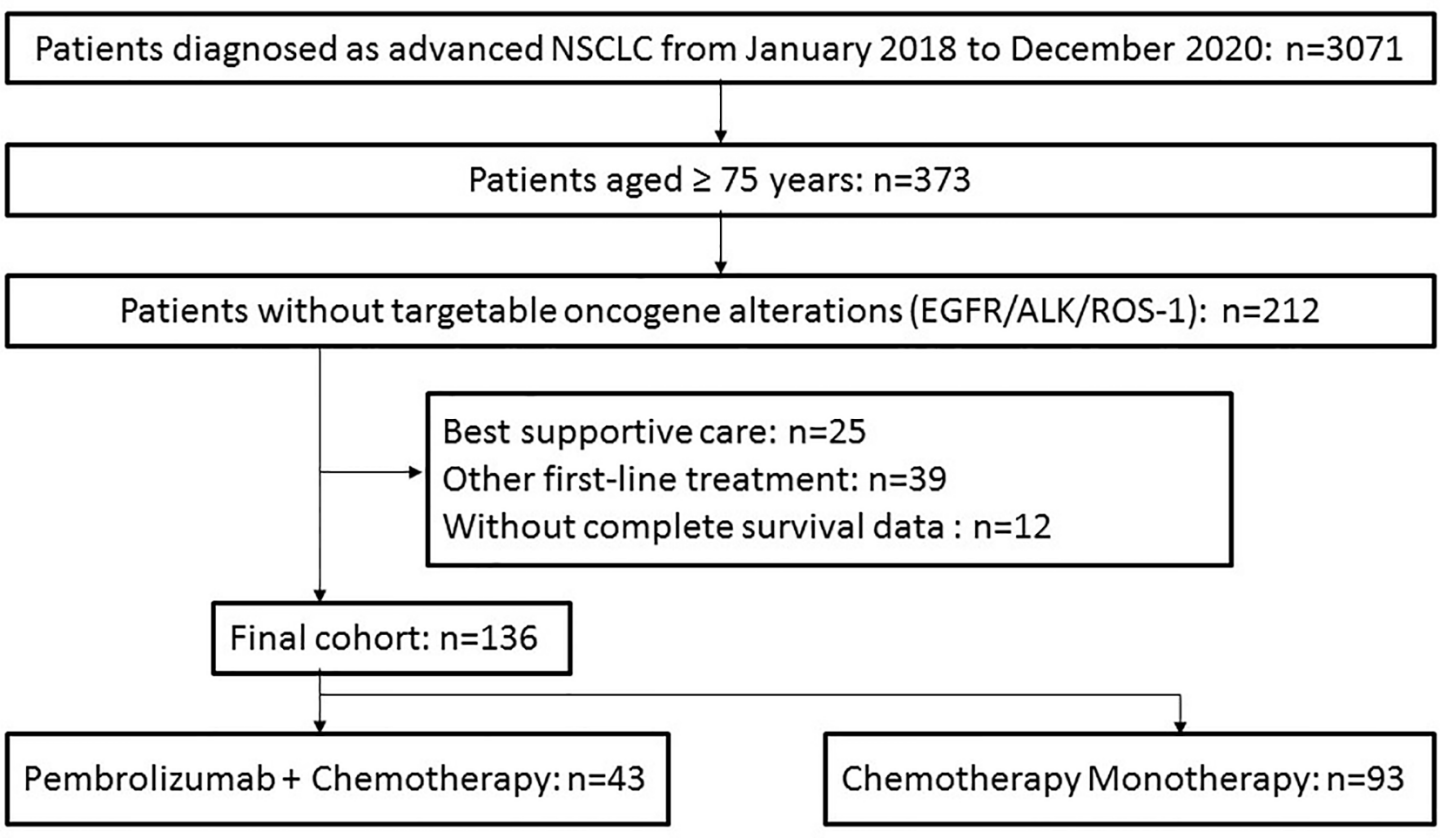
Flow diagram of patient selection steps.

Among 373 elderly patients diagnosed as advanced NSCLC from January 2018 to December 2020 in our hospital, 212 were tested without targetable oncogene alterations (EGFR/ALK/ROS-1) (**Figure 1**). After excluding 25 patients receiving best supportive care, 39 receiving other regimens (such as pembrolizumab monotherapy, chemotherapy plus bevacizumab, other immunotherapy) as first-line treatment, and 12 without complete survival data, 136 patients were included in our analysis finally. Among them, 93 patients were treated with chemotherapy monotherapy (CM), while the other 43 cases received pembrolizumab plus chemotherapy (P+C).

There was no significant difference between the two groups in age, gender, TNM stage, ECOG-PS, histology, and comorbidity (**Table 1**). In contrast, there were significantly more PD-L1 positive (TPS ≥ 1%) patients in the P+C group (46% vs. 24%), while patients with PD-L1 negative (TPS < 1%) (28% vs. 34%) and unknown PD-L1 (26% vs. 42%) were more in the CM group.

**Table 1:** Baseline characteristics of patients with different treatments before and after matching.

Characteristic	P+C (n=43) (%)	CM (n=93) (%)	*P*
**Age, years (range)**	77.9 (75–85)	77.3 (75–88)	0.23
**Gender**			0.37
**Male**	36 (84)	83 (89)	
**Female**	7 (16)	10 (11)	
**TNM stage**			0.38
**III**	21 (49)	53 (57)	
**IV**	22 (51)	40 (43)	
**Performance status (ECOG-PS)**			0.63
**0-1**	39 (91)	88 (95)	
**2-3**	4 (9)	5 (5)	
**Histology**			0.79
**Squamous cell carcinoma**	22 (52)	44 (47)	
**Adenocarcinoma**	16 (38)	37 (40)	
**Not otherwise specified**	4 (10)	12 (13)	
**PD-L1 TPS**			0.024
**<1%**	12 (28)	32 (34)	
**≥1%**	20 (46)	22 (24)	
**Unknown**	11 (26)	39 (42)	
**Simplified comorbidity score**			0.44
**>9**	6 (14)	18 (19)	
**≤9**	37 (86)	75 (81)	

### Survival Analysis

In chemotherapy monotherapy and combination therapy group, the objective response rate was 29% and 53% (P=0.006), and the disease control rate was 87% and 95%, respectively(P=0.24). Up to the final follow-up, 87 of 93 patients (94%) in CM group and 25 of 43 patients (58%) in P+C group had disease progression on first-line treatment. At the same time, 45 of 93 patients (48%) in CM group and 31 of 43 patients (72%) in the P+C group were still alive.

**Figure 2:**
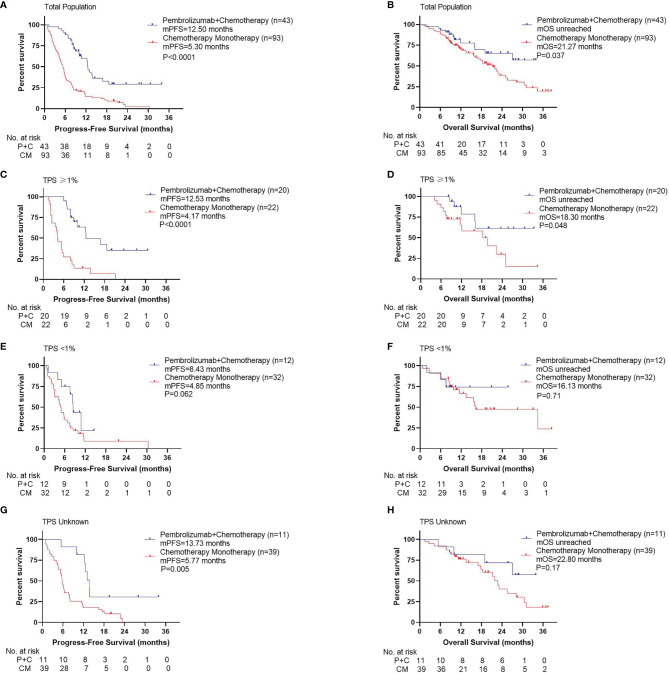
Survival curves for different treatments. **(A)** Progression-free survival, **(B)** Overall survival by treatments of total population. **(C)** Progression -free survival, **(D)** Overall survival by treatments of patients with TPS ≥1%. **(E)** Progression -free survival, **(F)** Overall survival by treatments of patients with TPS <1%. **(G)** Progression -free survival, **(H)** Overall survival by treatments of patients with TPS unknown.

Compared with patients in the CM group, patients in the P+C group had significantly longer PFS (**Figure 2A**, 12.50 months vs. 5.30 months, P<0.001). OS was also significantly longer in the P+C group (**Figure 2B**, unreached vs. 21.27 months, P=0.037). In subgroup analysis by PD-L1 TPS, we found that in patients with TPS≥1%, PFS and OS in P+C group were significantly longer than those in CM group (PFS: **Figure 2C**, 12.53 months vs. 4.17 months, P<0.001; OS: **Figure 2D**, unreached vs. 18.30 months, P=0.048). However, in patients with TPS<1%, the PFS and OS benefit was not significant in the P+C group (PFS: **Figure 2E**, 8.43 months vs. 4.85 months, P=0.062; OS: **Figure 2F**, unreached vs. 16.13 months, P=0.71). In patients with unknown TPS, PFS showed significant difference while OS did not (PFS: **Figure 2G**, 13.73 months vs. 5.77 months, P=0.005; OS: **Figure 2H**, unreached vs. 22.80 months, P=0.17).

**Figure 3:**
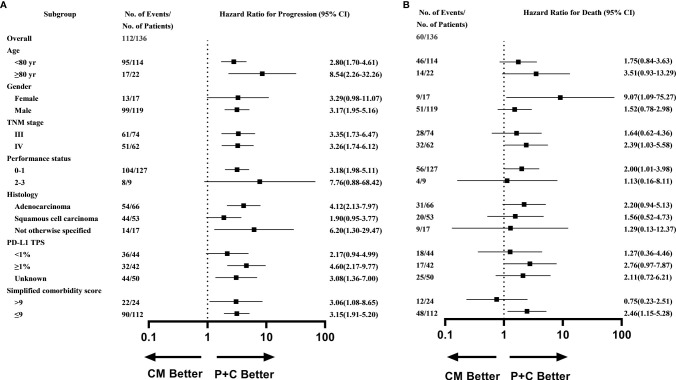
Subgroup analysis of **(A)** Progression-free survival **(B)** Overall survival in P+C and CM groups.

What’s more, subgroup analysis showed that most subgroups could access an evident PFS benefit with P+C (**Figure 3A**), except for patients with poor performance score (≥2), women, and patients with squamous cell carcinoma. While the subgroup analysis of OS showed that patients with TPS >1% (HR=2.76, 95%CI 0.97-7.87), stage IV (HR=2.39, 95%CI 1.03-5.58), good performance score (ECOG-PS <2) (HR=2.00, 95%CI 1.01-3.98), fewer comorbidities (SCS <9) (HR=2.46, 95%CI 1.15-5.28) or female patients (HR=9.07, 95%CI 1.09-75.27) could benefit more significantly from P+C (**Figure 3B**).

In multivariable analyses, the treatment regimen (PFS: P<0.001, HR=3.34, 95%CI 2.07-5.38; OS: P=0.005, HR=2.62, 95%CI 1.34-5.13) and comorbidities (PFS: P=0.033, HR=1.69, 95%CI 1.04-2.73; OS: P=0.020, HR=2.19, 95%CI 1.13-4.26) had significant effects on both PFS and OS (**Table 2**). What’s more, elder age (≥80years) (P=0.016, HR=2.17, 95%CI 1.16-4.08) and higher TNM stage (P=0.010, HR=2.01, 95%CI 1.18-3.43) had a negative effect on OS.

Table 2AUnivariable and multivariable analysis on disease-free survival.

**Table:** 

Variable	Univariable analysis		Multivariable analysis	
	HR (95%CI)	P	HR (95%CI)	*P*
**Age**				
**<80 yr**	Ref			
**≥80 yr**	0.96 (0.57-1.62)	0.88		
**Gender**				
**Female**	Ref			
**Male**	1.14 (0.85-1.52)	0.39		
**TNM stage**				
**III**	Ref			
**IV**	1.12 (0.77-1.63)	0.56		
**Performance status**				
**0-1**	Ref			
**2-3**	1.05 (0.52-2.16)	0.89		
**Treatment**				
**P+C**	Ref		Ref	
**CM**	3.19 (2.03-5.01)	<0.001	3.34 (2.07-5.38)	<0.001
**Histology**				
**Adenocarcinoma**	Ref			
**Squamous cell carcinoma**	1.16 (0.78-1.73)	0.46		
**Not otherwise specified**	1.54 (0.84-2.85)	0.16		
**PD-L1 TPS**				
**<1%**	Ref		Ref	
**≥1%**	0.64 (0.40-1.04)	0.072	0.78 (0.50-1.22)	0.28
**Unknown**	0.75 (0.48-1.17)	0.20	0.95 (0.58-1.56)	0.83
**Simplified comorbidity score**				
**≤9**	Ref		Ref	
**>9**	1.65 (1.02-2.64)	0.040	1.69 (1.04-2.73)	0.033

**Table 2B:** Univariable and multivariable analysis on overall survival.

Variable	Univariable analysis		Multivariable analysis	
	HR (95%CI)	*P*	HR (95%CI)	*P*
**Age**				
**<80 yr**	Ref		Ref	
**≥80 yr**	1.87 (1.02-3.42)	0.042	2.17 (1.16-4.08)	0.016
**Gender**				
**Female**	Ref			
**Male**	1.01 (0.71-1.44)	0.96		
**TNM stage**				
**III**	Ref		Ref	
**IV**	1.69 (1.01-2.81)	0.045	2.01 (1.18-3.43)	0.010
**Performance status**				
**0-1**	Ref			
**2-3**	0.89 (0.32-2.45)	0.82		
**Treatment**				
**P+C**	Ref		Ref	
**CM**	1.94 (1.03-3.67)	0.041	2.62 (1.34-5.13)	0.005
**Histology**				
**Adenocarcinoma**	Ref			
**Squamous cell carcinoma**	1.41 (0.81-2.48)	0.23		
**Not otherwise specified**	1.79 (0.81-3.95)	0.15		
**PD-L1 TPS**				
**<1%**	Ref			
**≥1%**	0.88 (0.45-1.71)	0.70		
**Unknown**	0.83 (0.45-1.53)	0.83		
**Simplified comorbidity score**				
**≤9**	Ref		Ref	
**>9**	1.88 (0.99-3.57)	0.055	2.19 (1.13-4.26)	0.020

### Safety and Toxicity Profile

We analyzed reasons for discontinuing first-line therapy in two groups (**Table 3A**). In P+C group, 26% of patients discontinued due to adverse events (AEs) and 44% due to disease progression, compared with 5% and 88% in the CM group, respectively. However, there was no significant difference in deaths due to AEs between the two groups (5% vs. 4%, P=0.93). Of the 11 patients in the P+C group who discontinued due to AEs, 6 were due to pneumonia of any grade and 1 each to thrombocytopenia, myocarditis, hypothyroidism, skin rash and anemia. On the other hand, whether at 3, 6 or 12 months of treatment, more patients remained on first-line treatment in the P+C group than in the CM group.

**Table 3A:** Reasons of discontinuation in different treatment groups.

	End of follow-up	At 3 months	At 6 months	At 12 months
**P+C (n=43)**				
**Continuation of first-line treatment**	11 (26)	40 (93)	32 (74)	20 (47)
**Discontinuation due to PD**	19 (44)	1 (2)	4 (9)	11 (26)
**Discontinuation due to AEs**	11 (26)	2 (5)	5 (12)	10 (23)
**Discontinuation due to others**	2 (5)	0 (0)	2 (5)	2 (5)
**Death due to AEs**	2 (5)	0 (0)	1 (2)	2 (5)
**CM (n=93)**				
**Continuation of first-line treatment**	5 (5)	68 (73)	31 (33)	11 (12)
**Discontinuation due to PD**	82 (88)	23 (25)	56 (60)	76 (82)
**Discontinuation due to AEs**	6 (6)	2 (2)	6 (6)	6 (6)
**Discontinuation due to others**	0 (0)	0 (0)	0 (0)	0 (0)
**Death due to AEs**	4 (4)	2 (2)	4 (4)	4 (4)

We then collected adverse events during treatment in two groups (**Table 3B**). There was no difference in the incidence of serious AEs (grade 3 or higher) between the two groups, which was 67% and 61% in P+C and CM group, respectively (P=0.49). The most common AEs in both groups were neutropenia, thrombocytopenia, and anemia. The incidence of most adverse events did not differ significantly between the two groups, except pneumonia (9% vs. 0%, P=0.009) and dyspnea (7% vs. 1%, P=0.093), which were more common in the P+C group.

**Table 3B:** Grade ≥3 adverse events in different treatment groups.

	P+C (n=43) (%)	CM (n=93) (%)	*P*
**Any events**	29 (67)	57 (61)	0.49
**Neutropenia**	6 (14)	18 (19)	0.44
**Thrombocytopenia**	5 (12)	10 (11)	0.88
**Anemia**	4 (9)	12 (13)	0.54
**ALT increased**	2 (5)	3 (3)	0.69
**Skin rash**	1 (2)	2 (2)	0.95
**Anorexia**	2 (5)	4 (4)	0.93
**Nausea**	2 (5)	3 (3)	0.69
**Diarrhea**	1 (2)	2 (2)	0.95
**Constipation**	0 (0)	1 (1)	0.50
**Fatigue**	4 (9)	6 (6)	0.81
**Fever**	2 (5)	2 (2)	0.59
**Hypothyroid**	2 (5)	0 (0)	0.18
**Dyspnea**	3 (7)	1 (1)	0.093
**Pneumonia**	4 (9)	0 (0)	0.009
**Myocarditis**	1 (2)	0 (0)	0.32

### Treatment After Disease Progression

**Supplementary Table 1** listed the secondary therapies initiated in 25 patients of P+C group and 87 patients of CM group after failure of first-line treatments. Best supportive care without second-line therapy was chosen in 12 patients in the P+C group and 19 in the CM group, respectively. A total of 20 CM patients were treated with immune checkpoint inhibitors in the second line, with or without other treatments; 2 patients in the P+C group underwent immunotherapy re-challenge with nivolumab and 4 received pembrolizumab plus bevacizumab. Anlotinib, an oral small-molecular multi-targeting tyrosine kinase inhibitor, was selected by 3 patients in the P+C group and 11 patients in the CM group. Local treatment, including radiotherapy and ablation, was administered in 7 patients.

## Discussion

For patients with advanced non-small-cell lung cancer (NSCLC) without targetable oncogene alterations (*EGFR*, *ALK*, *ROS-1*, etc.), chemotherapy used to be the best first-line treatment. However, immune checkpoint inhibitors (ICIs), especially those targeting the programmed cell death protein (PD-1) and its ligand (PD-L1), have revolutionized this situation (1, 4). Several trials have demonstrated the efficacy of pembrolizumab (a PD-1 inhibitor) in combination with chemotherapy in patients with advanced NSCLC, regardless of tumor PD-L1 expression (7, 8, 14).

The phase 2 KEYNOTE-021 cohort G first showed a higher benefit of P+C compared to chemotherapy monotherapy (CM) in patients with advanced NSCLC (PFS: 24.5 months vs. 9.9 months; OS: 34.5 months vs. 21.1 months) (9). KEYNOTE 189 and KEYNOTE 407 further demonstrated that compared to CM, P+C significantly improved PFS and OS in patients with metastatic non-squamous and squamous NSCLC, respectively (non-squamous: PFS: 9.0 months vs. 4.9 months; OS: 22.0 months vs. 10.7 months; squamous: PFS: 8.0 months vs. 5.1 months; OS: 17.1 months vs. 11.6 months) (7, 8).

Due to the aging population, the incidence of NSCLC in the elderly (≥75 years old) is increasing (15, 16). Nevertheless, elderly patients are often excluded from clinical trials because of their reduced ability to tolerate treatment (e.g., performance score [ECOG PS] ≥2), comorbidities, and potential differences in drug metabolism (17, 18). A recent study showed that elderly patients receiving first-line P+C had significantly shorter PFS and OS than younger patients (PFS: 6.2 months vs. 9.7 months, P= 0.004; OS: 11 months vs. not reached, P<0.001) (19). Elderly patients in our cohort had longer PFS and OS, possibly because there were more stage III patients.

In our cohort, about half of elderly NSCLC patients were PD-L1 negative, and a proportion of patients did not undergo PD-L1 testing. However, the results of subgroup analysis showed no significant difference in OS between these patients using P+C and CM. Meanwhile, some subgroups, including male patients, patients with stage III, PS score ≥2, squamous cell carcinoma and undetermined pathology, simple comorbidity score > 9, did not benefit significantly from P+C. This might be related to the small sample size of our study, but it also reflected that P+C was more suitable for elderly patients with metastatic disease or a better performance status.

Safety was another topic of concern for elderly patients. Age-related physiological changes and comorbidities may increase treatment-related toxicities and reduce tolerance to therapy (20, 21). Compared with chemotherapy, pembrolizumab monotherapy was associated with fewer treatment-related AEs and fewer discontinuations (4, 22, 23). Subgroup analysis of elderly patients showed advanced age was not associated with increased toxicity with pembrolizumab (24). For P+C, data from clinical trials showed patients could benefit from the combination therapy without additional toxicity compared to CM (7, 8). In this study, the discontinuation rate in elderly patients (26%) was slightly higher to that of general population in previous studies (16.2% in Keynote407, 17% in Keynote021-G) (8, 9), possibly because AEs had a more significant impact on quality of life and treatment, leading to higher discontinuation of treatment even when no serious toxicity occurred (12, 25).

Our research has the following limitations. First, as a retrospective single-center study, loss of follow-up and selective bias in the treatment were inevitable. However, patients were enrolled consecutively and adjusted using multivariable analysis to reduce the bias. Second, because AEs were only collected from medical records, we simply analyzed AEs of grade 3 or higher. Further studies are needed to analyze the safety of P+C in elderly patients in more detail.

In conclusion, our study evaluated first-line therapy for elderly patients with advanced NSCLC without targetable oncogene alterations. Elderly patients using P+C were more likely to discontinue due to adverse effects, so disease stage, PD-L1 expression, performance score and comorbidities should be considered when selecting first-line treatment.

## Data Availability Statement

The raw data supporting the conclusions of this article will be made available by the authors, without undue reservation.

## Ethics Statement

The studies involving human participants were reviewed and approved by institutional review board of Shanghai Chest Hospital. The patients/participants provided their written informed consent to participate in this study.

## Author Contributions

ZY, YC, and YW all have substantial contributions to the conception or design of the work, the collection and analysis of data, the writing and edit of the article. The rest authors have given substantial contributions to the work by providing editing and writing assistance. All authors contributed to the article and approved the submitted version.

## Funding

This work was supported by the foundation of Shanghai Chest Hospital (Project No. YJXT20190102); the program of system biomedicine innovation center from Shanghai Jiao Tong University (Project No. 15ZH4009); Shanghai Jiao Tong University School of Medicine (Project No. 15ZH1008) and the foundation of Chinese society of clinical oncology (Project No. Y2019AZZD-0355).

## Conflict of Interest

The authors declare that the research was conducted in the absence of any commercial or financial relationships that could be construed as a potential conflict of interest.

## Publisher’s Note

All claims expressed in this article are solely those of the authors and do not necessarily represent those of their affiliated organizations, or those of the publisher, the editors and the reviewers. Any product that may be evaluated in this article, or claim that may be made by its manufacturer, is not guaranteed or endorsed by the publisher.

## Supplementary Material

Supplementary Table 1 Treatments after disease progression in different treatment groups.
