# Supplementary material for: Pembrolizumab Plus Chemotherapy Versus Chemotherapy Monotherapy as a First-Line Treatment in Elderly Patients (≥75 Years Old) With Non-Small-Cell Lung Cancer
Source: Front Immunol. 2022 Feb 14;13:807575. doi: 10.3389/fimmu.2022.807575 (PMC8882651; doi:10.3389/fimmu.2022.807575)
Supplement: Supplementary Table 1 — Treatments after disease progression in different treatment groups. [file Table_1.docx]

Supplement Table 1.

Treatments after disease progression in different treatment groups.

| Treatment | P+C (n=25) | CM (n=87) |
| --- | --- | --- |
| Best supportive care | 12 | 19 |
| Chemotherapy±Bevacizumab | 3 | 31 |
| Immunotherapy alone | 2 | 14 |
| Immunotherapy+Bevacizumab | 4 | 3 |
| Immunotherapy+Chemotherapy | 0 | 3 |
| Anlotinib | 3 | 11 |
| Local treatment | 1 | 6 |
